# 2024 Bangladesh Floodwaters Harbour Regional Hotspots of Pathogens and Antimicrobial Resistance

**DOI:** 10.1111/1758-2229.70240

**Published:** 2025-11-19

**Authors:** Tanzim Rahman, Nure Sharaf Nower Samia, Shovan Basak Moon, Md. Shafiqul Islam, Zahid Hayat Mahmud, Md. Shahidul Kabir, Muhammad Manjurul Karim, Mustafizur Rahman, Mohammad Jubair

**Affiliations:** ^1^ Laboratory of Environmental Health, Health Systems and Population Studies Division International Centre for Diarrhoeal Disease Research, Bangladesh (icddr,b) Dhaka Bangladesh; ^2^ Genome Centre, Infectious Diseases Division International Centre for Diarrhoeal Disease Research, Bangladesh (icddr,b) Dhaka Bangladesh; ^3^ Department of Microbiology Notre Dame University Bangladesh Dhaka Bangladesh; ^4^ Department of Microbiology University of Dhaka Dhaka Bangladesh

**Keywords:** AMR, Bangladesh, environmental microbiology, floodwaters, metagenomics, pathogens, public health

## Abstract

Seasonal flooding in Bangladesh poses severe public health risks through waterborne disease, yet a comprehensive, genomic‐level understanding of the associated microbial hazards is lacking. This study presents a comprehensive shotgun metagenomic analysis of floodwaters from four districts of Bangladesh (Cumilla, Feni, Lakshmipur, and Noakhali) during the devastating 2024 floods, profiling the distribution of pathogenic bacteria, antimicrobial resistance genes, and virulence factors. A total of 12 samples were collected during peak flooding periods and processed using Illumina sequencing. Taxonomic profiling and resistome analysis were performed using MetaPhlAn4, ABRicate, and MEGAHIT, referencing the NCBI and CZ ID databases. Across all regions, 301 operational taxonomic units were identified. Feni exhibited the highest diversity of pathogenic species, including multidrug‐resistant *Klebsiella pneumoniae* , toxigenic *Pseudomonas aeruginosa* , and mobilizable resistance plasmids (e.g., IncP1, Col440I). Noakhali samples revealed co‐detection of zoonotic and emerging pathogens such as *Aliarcobacter* spp. and *Streptococcus suis* , along with key resistance genes like *blaOXA* and *ermB*. Microbial community clustering revealed strong spatial heterogeneity. This study provides genomic evidence that floodwaters harbour emerging pathogens and AMR. It strongly advocates for incorporating metagenomic tools into Bangladesh's national flood response and AMR monitoring frameworks.

## Graphical abstract

Metagenomic analysis of Bangladesh floodwaters reveals diverse pathogens ( *Klebsiella pneumoniae* , *Pseudomonas aeruginosa* ), antimicrobial resistance genes (*blaOXA*, *ermB*), and mobilizable plasmids (IncP1, Col440I), highlighting floodwaters as reservoirs for microbial hazards and AMR dissemination, with region‐specific risks demanding targeted public health interventions.

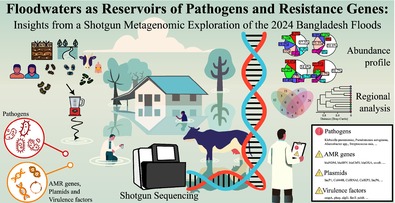

## 1 Introduction

Water‐borne illnesses pose a serious threat to public health (Rajina et al. 2024; Kunz et al. 2024). This is particularly true in regions with poor sanitation and limited healthcare facilities. Research indicates that hospitalisation rates due to bacterial pathogen groups is 16% lower in urban areas compared to rural locations (Lynch and Shaman 2024), highlighting a disparity in healthcare access and disease burden. A lack of access to safe drinking water further contributes to high mortality rates (Manetu and Karanja 2021; Huang et al. 2024), especially during floods when clean water sources become contaminated (Jerin et al. 2024). Water‐borne pathogens, including bacteria such as *Vibrio cholerae* and *Escherichia coli* , and viruses such as hepatitis A and rotavirus, can cause a range of diseases, such as diarrhoea, dysentery, cholera, and hepatitis. According to the World Health Organization (WHO), amongst children younger than 5 years of age, diarrhoea is the third leading cause of death, killing almost 450,000 each year (World Health Organization 2024). Risk factors of diarrheal mortality include unsafe water storage and a lack of water treatment and sanitation practises (Mebrahtom et al. 2022; Manetu et al. 2021), particularly in low and middle‐income countries (Manetu et al. 2021; Hartman et al. 2022).

Bangladesh is located on the world's largest delta, where an extensive network of rivers flows through a significant portion of the country. Its position in the tropical monsoon climatic region results in heavy rainfall for most of the year, particularly during the monsoon months of June to October (Mandal et al. 2020; Abdullah et al. 2021), which along with the low‐lying flat topology of the country leads to frequent widespread flooding along the riverbanks (Rana et al. 2024). Uddin and Matin (2021) identified 13% of Bangladesh as highly flood‐prone, whilst Zarjes et al. (Kader et al. 2024) classified flood susceptibility into five categories, finding 16% of the country in the ‘very high’ and 42.8% in the ‘high’ susceptibility classes. As a low‐income country with limited access to adequate health infrastructure, especially in impoverished regions (Alamgir et al. 2020), Bangladesh faces a significant challenge to health care delivery with Hasan et al. stating that the country spent 4% of its GDP per capita on diarrheal treatment (Hasan et al. 2021). Poor sanitation and insufficient access to clean water (Shackleton et al. 2023; Khan et al. 2022) contribute to a high prevalence of water‐borne diseases (Tadesse et al. 2023; Nguyen et al. 2024; Waddington et al. 2023; Yeasmin et al. 2022). Consequently, one of the leading causes of mortality in the country is from diarrheal diseases (Nguyen et al. 2024), specifically targeting infants (Yeasmin et al. 2022; Hossain et al. 2022; Rahman and Hossain 2022), leading to a critical demand for robust public health monitoring systems. Early detection of outbreaks is essential to prevent large‐scale infections that may overwhelm the already strained health infrastructure.

Metagenomics involves the direct recovery and analysis of genetic material from environmental samples—such as soil, water, or biological matrices—without the requirement to culture individual organisms (Thomas et al. 2012; Zhang et al. 2021). By sequencing the collective DNA, researchers can generate comprehensive profiles of microbial community composition, diversity, and functional potential (Liu et al. 2022). This capability gives metagenomics considerable promise as a tool for health surveillance, able to detect microbial signatures in floodwater indicative of public health threats (Kim et al. 2022). In particular, taxonomic profiles derived from metagenomic data can be scanned for known pathogens and used to infer antimicrobial resistance, enabling early detection of disease outbreaks and assessment of emerging health risks (Miller et al. 2013).

Floodwater metagenomics is now vital for evaluating microbial contamination and public health threats. Globally, studies such as Kim et al. have demonstrated the effectiveness of metagenomic sequencing in identifying pathogens and antimicrobial resistance (AMR) genes in flood‐affected regions, revealing that floodwaters following drought periods pose higher public health risks compared to those after regular rainfall events (Kim et al. 2022). Further supporting this, Sharma et al. employed metagenomics to analyze microbial communities and AMR genes in river sediments, highlighting the role of flood‐induced contamination in disseminating antibiotic resistance, particularly in environments with poor sanitation (Sharma et al. 2024). Their findings align with observations in the Ganges–Brahmaputra Delta, where seasonal flooding correlates with shifts in pathogenic bacterial populations. Despite these advances, significant gaps persist in the real‐time, high‐resolution genomic monitoring of pathogens and AMR determinants during acute flooding events, especially in resource‐limited settings like Bangladesh (Van Poelvoorde et al. 2025; Getchell et al. 2024). Previous studies have largely focused on river sediments (Sharma et al. 2024) or post‐drought flooding scenarios in other geographical contexts (Kim et al. 2022); a comprehensive shotgun metagenomic survey of floodwaters during a catastrophic monsoon event in the Ganges–Brahmaputra Delta has not been conducted.

In August 2024, Bangladesh experienced catastrophic flooding, particularly in eastern regions, due to unprecedented monsoon rainfall. This crisis was intensified by political transitions that hindered emergency response efforts, as well as cross‐border water release from Indian dams (The Daily Star 2024). The catastrophic flooding affected approximately 5.8 million individuals, resulting in 71 fatalities and leaving 582,155 families stranded amid widespread infrastructure destruction (International Federation of Red Cross and Red Crescent Societies 2024). These conditions precipitated significant water supply contamination, heightening risks of pathogenic outbreaks and accelerated antimicrobial resistance (AMR) dissemination in affected regions. This study builds upon previous research by applying shotgun metagenomics to the 2024 Bangladesh floods, specifically aiming to: (1) provide a high‐resolution snapshot of the pathogenic and AMR landscape; and (2) identify regionally distinct microbial diversity that reflects local environmental and anthropogenic factors.

## 2 Materials and Methods

### 2.1 Sample Collection

**FIGURE 1:**
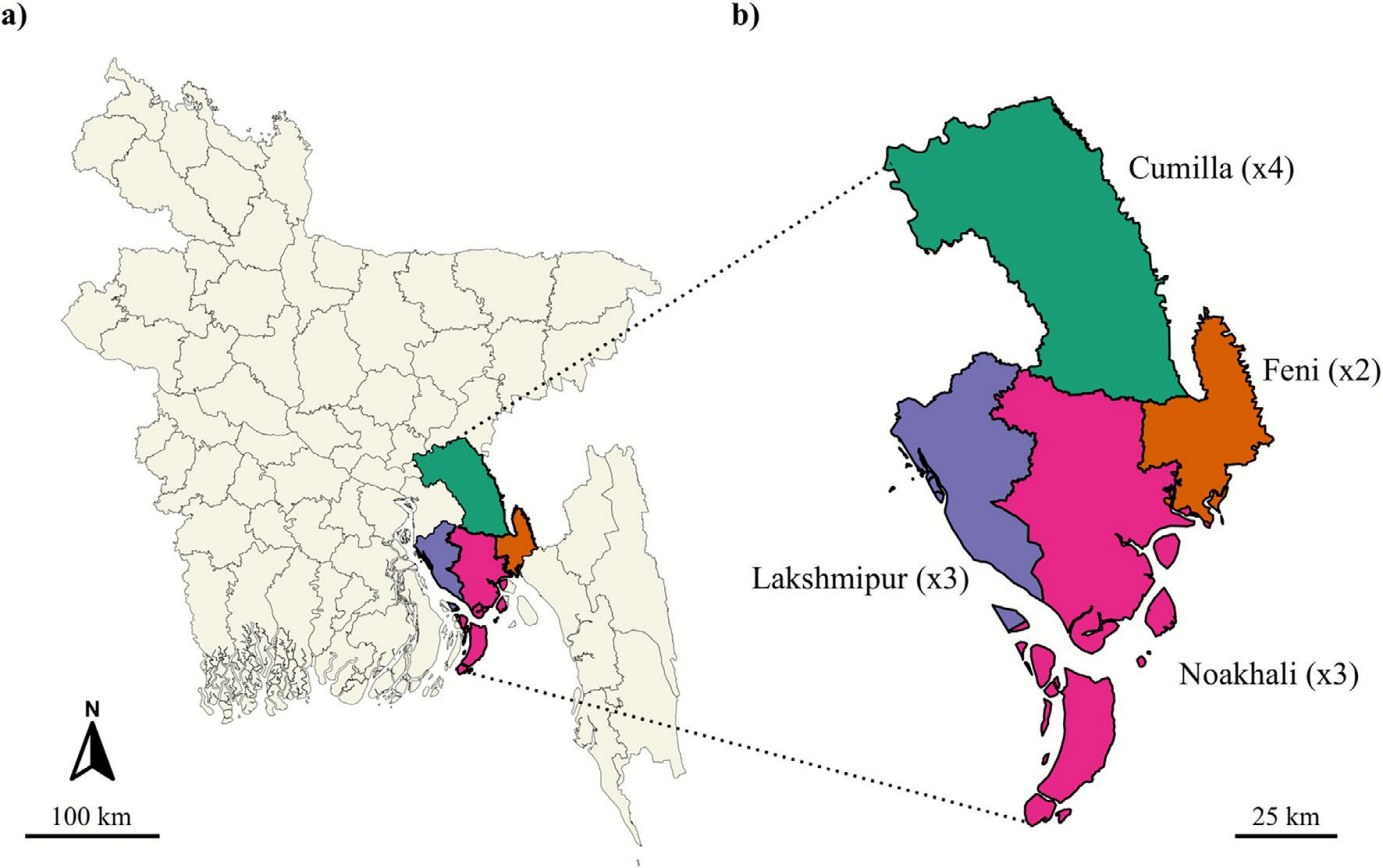
(a) Map of Bangladesh showing the four districts where floodwater samples were collected, each highlighted in a different colour. (b) Enlarged view of the selected districts and the number of samples collected indicated in parentheses.

The south‐eastern parts of the country, specifically the districts of Cumilla, Feni, Lakshmipur and Noakhali, were chosen as the sampling location due to particularly intense flooding in the region during the 2024 Bangladesh flood event. Flood water samples were collected according to the EPA guidelines for water sampling (https://www.epa.gov/sites/default/files/2015‐11/documents/drinking_water_sample_collection.pdf). A total of 12 samples were collected: four from Cumilla, two from Feni, and three each from Lakshmipur and Noakhali (Figure 1). The sampling strategy was designed to cover the affected regions, but was constrained by the flood conditions, which limited access to remote areas.

Within each district, samples were collected from distinct, accessible sites to capture local variability. Due to the logistical challenges of the flood response and the remote nature of the sites, it was not feasible to maintain a portable cooler or collect formal triplicate samples. Instead, a single, representative 1‐L sample was collected from each distinct location. At each site, surface water samples (approximately 0.5 m depth) were collected in sterile, DNA‐free 1‐L bottles. Bottles were filled whilst submerged with the opening facing the current to ensure a representative sample and minimise contamination from surface debris. Samples were transported at ambient temperature and processed for filtration within 12 h of collection to minimise microbial community changes.

### 2.2 DNA Extraction

For DNA extraction, 1000 mL of flood water was filtered through a 0.22 μm nitrocellulose membrane (G‐Biosciences, 786056NC) to capture microbial biomass as in similar environmental DNA studies (Wu et al. 2024). Depending on turbidity, 4–5 or more membranes were used, each filtering a minimum of 100 mL of water. The procedure was repeated until the entire volume was processed. After filtration, all the membranes containing retained microbial biomass were resuspended in 800 μL of phosphate‐buffered saline (PBS, pH 7.2–7.4; Thermo Fisher,10,010,023) to recover microorganisms for DNA extraction. PBS was used as it maintains isotonic and physiological conditions, preventing osmotic lysis and preserving cell integrity during recovery (Miller et al. 2013). Total genomic DNA was then extracted from the concentrated solution using the DNeasy Blood & Tissue Kit (Qiagen, 69,504) following the manufacturer's instructions, with modifications adapted from the CDC PulseNet Total DNA Extraction Protocol (https://www.aphl.org/programs/global_health/Documents/PNL33_DNA_Extraction_and_Quality.pdf). DNA purity and concentration were assessed using a NanoDrop spectrophotometer (Thermo Fisher Scientific, ND 1000) and a Qubit 4.0 Fluorometer (Thermo Fisher Scientific, Cat. No. Q33238), respectively. High‐quality DNA was confirmed by a 260/280 ratio of ~1.8 and a 260/230 ratio of ~2.0.

### 2.3 Library Preparation and Sequencing

Library preparation was performed using the Illumina DNA Prep Kit (Illumina, 20,060,059) on an automated epMotion 5075 liquid handler (Eppendorf, Hamburg, Germany) to ensure reproducibility and minimise cross‐contamination. Post‐pooling, library concentration was quantified using a Qubit 4.0 Fluorometer (Thermo Fisher Scientific, Q33238) with the Qubit dsDNA HS Assay Kit (Thermo Fisher Scientific, Q32851), confirming adequate yields for sequencing. Following quantification, pooled libraries were denatured and diluted according to the NextSeq 500/550 System Denature and Dilute Libraries Guide (Illumina, Document No. 15048776). A final concentration of 1.5 pM was loaded onto the NextSeq 500 sequencing platform (Illumina, San Diego, CA, USA) using a NextSeq 500/550 High Output Kit v2.5 (150 cycles; Illumina, 20024906). Sequencing was performed with paired‐end 2 × 150 bp reads, employing fluorescently labelled dNTPs for base detection, to achieve high‐resolution genomic coverage.

### 2.4 Data Analysis

Following shotgun metagenomic sequencing of floodwater samples, a comprehensive bioinformatics workflow was implemented to characterise microbial community composition, and identify potential pathogens, through the detection of AMR genes, plasmids, and virulence factors.

### 2.5 Preprocessing and Quality Control

The machine‐generated reads often contain imperfections that may impact the accuracy of downstream analyses. To address this, several preprocessing steps were performed. The first of these involved using the tool *bcl2fastq*, which demultiplexed the sequence data and converted the base call (BCL) files to the much more versatile FastQ format.

In order to enhance the quality of the reads, *FastP* (Chen 2023; Chen et al. 2018) was employed to perform adapter trimming and quality filtering. The filtered reads were then processed using *Bowtie2* (Langmead and Salzberg 2012), which maps sequences against a database, in order to filter out host‐origin sequences. These preprocessing steps produced high‐quality read pairs which were subsequently used for taxonomic profiling and sequence assembly.

### 2.6 Taxonomic Profiling and Statistical Analysis

Taxonomic profiling of the floodwater samples was conducted using *MetaPhlAn4* (Blanco‐Míguez et al. 2023), generating a high‐resolution abundance table consisting of Operational Taxonomic Units (OTUs) for each sample. These OTU tables, which reflect the relative abundance of microbial taxa, were subsequently merged into a comprehensive dataset encompassing all four regions. To facilitate downstream analysis, a custom Python script was developed to reformat the merged abundance data into three separate Excel sheets: an OTU matrix capturing the relative abundance of each OTU across all samples, a taxonomy table outlining the full taxonomic lineage of each OTU from kingdom to species, and a metadata sheet describing sample‐specific attributes. Using a custom R script, these structured datasets were integrated into a *Phyloseq* (McMurdie and Holmes 2013) object which was subsequently imported into the R package *microeco* (Liu et al. 2020), which provided a suite of tools for data visualisation and comparative analysis.

Visualisation outputs included both bar and pie charts illustrating the relative abundance of the most dominant OTUs in each sample. Additionally, in order to investigate the similarities and differences amongst the regional microbial communities, a Venn diagram was generated to highlight shared and unique OTUs amongst regions, whilst beta diversity analysis using the Bray–Curtis dissimilarity metric was employed to investigate regional clustering patterns. These analyses provided insights into microbial distribution and potential environmental influences shaping the microbiomes of different flood‐impacted regions.

### 2.7 Pathogen Screening

For pathogen screening, we cross‐referenced our OTUs against two widely recognised and standard databases for clinical and environmental pathogen detection: the National Center for Biotechnology Information (NCBI) Pathogen Detection database (National Center for Biotechnology Information, n.d.) and the Chan Zuckerberg ID (CZ ID) pathogen list (Chan Zuckerberg ID 2024). By cross‐referencing the OTUs detected in the samples with isolates listed in these databases, it was possible to directly identify exact matches as confirmed pathogens. Additionally, OTUs that showed close similarity to known pathogens, but did not match exactly, were flagged as potential pathogenic candidates. These flagged taxa could then be further investigated through literature review to assess their pathogenicity and relevance to public health risk.

### 2.8 AMR Genes, Plasmids and Virulence Factors

We utilised the tool ABRicate (Seemann 2020), which is specifically designed to screen assembled genomic sequences for resistance genes and other markers of concern. Since ABRicate operates only on assembled contigs rather than raw reads, initial read assembly was performed using MEGAHIT (Li et al. 2015, 2016), a fast and memory‐efficient assembler suited for metagenomic data. A minimum contig length of 1000 base pairs was set to exclude short sequences from the analysis which were likely to be non‐informative. The resulting contigs were then analysed with ABRicate using various databases: NCBI's AMRFinderPlus (Feldgarden et al. 2019) to identify AMR genes, PlasmidFinder (Carattoli and Hasman 2019) to detect plasmid sequences, and the Virulence Factors Database (VFDB) (Liu et al. 2021) to screen for bacterial virulence factors. As a result, a comprehensive view of the genetic elements contributing to both pathogenicity and resistance in the floodwater microbiome was achieved, offering critical insights into the potential health risks and aiding in the identification of high‐priority targets for further surveillance and intervention.

## 3 Results

### 3.1 Abundance Profile

**FIGURE 2:**
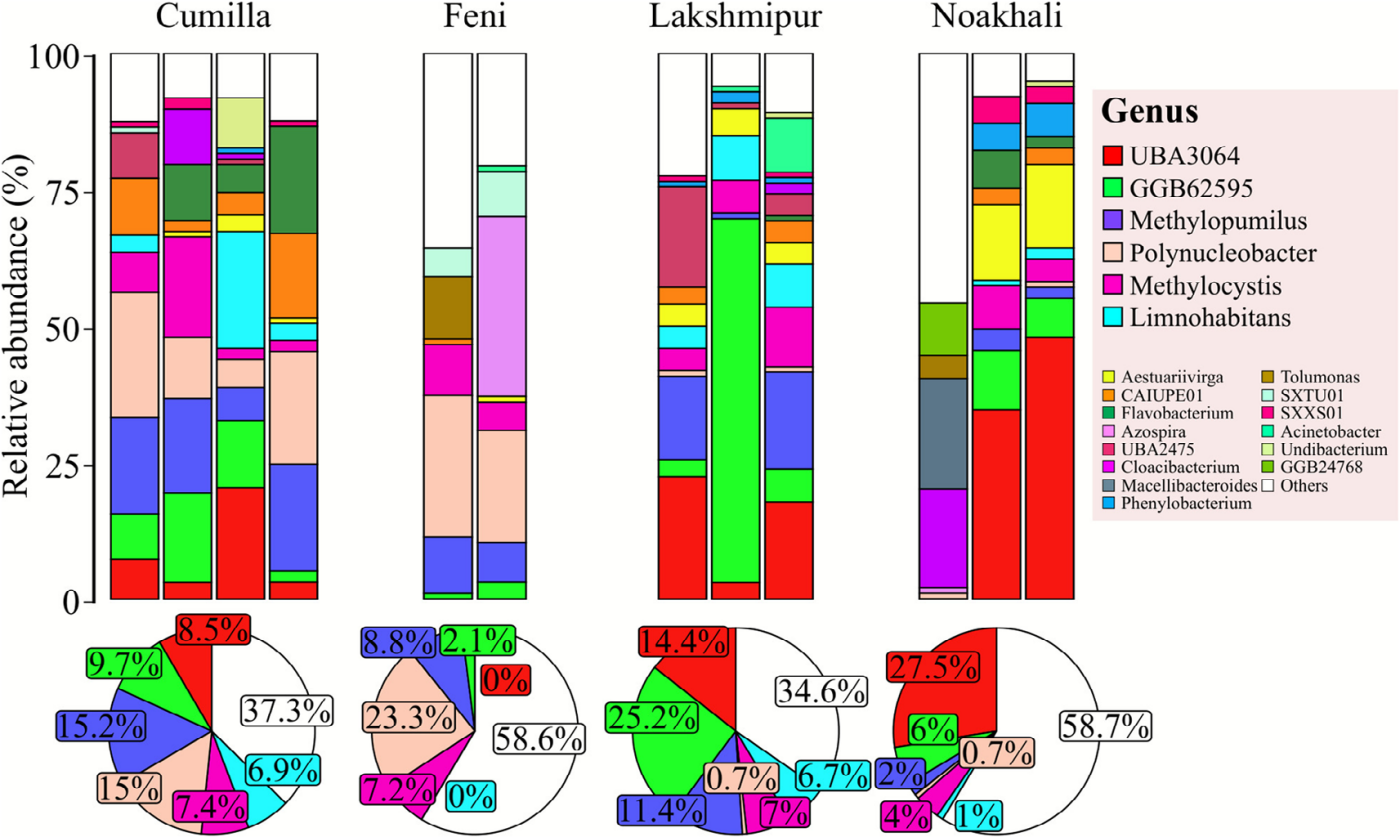
Top: Stacked bar plots showing the relative abundance profiles of the 20 most prevalent bacterial genera across all samples, grouped by district (Cumilla, Feni, Lakshmipur, and Noakhali). Distinct colour coding highlights inter‐district variation and the dominance of specific genera. Bottom: Pie charts illustrating the mean relative abundance of the six dominant genera in each district, revealing spatial heterogeneity in microbial community structure and highlighting differences in genus‐level dominance patterns across locations.

Analysis of the floodwater microbiome revealed diverse microbial communities that were structurally dominated by a limited number of non‐pathogenic, environmental genera, with distinct compositional patterns across the four districts. Taxa with less than 0.5% abundance across all samples were removed and 101 operational taxonomic units (OTUs) were analysed from a total of 301 initially identified. The overall structure and regional variations of these communities are visualised in Figure 2. A clear dominance of the phylum Proteobacteria was observed across all regions, constituting 70.7% of the community in Cumilla, 88.6% in Feni, 53.5% in Lakshmipur, and 66.3% in Noakhali. This proteobacterial dominance is further reflected in the pie charts, which illustrate the mean relative abundance of the top six genera for each district. The six most abundant genera—*UBA3064*, *GGB62595*, *Methylopumilus*, *Polynucleobacter*, *Methylocystis*, and *Limnohabitans*—collectively accounted for a substantial portion of each region's microbiome: 62.7% in Cumilla, 41.3% in Feni, 65.4% in Lakshmipur, and 41.2% in Noakhali. Notably, GGB62595, the second most abundant genus, is a Genus‐level Genome Bin (GGB) from the Gemmataceae family that could not be classified more precisely. Consistent with their taxonomic affiliation, all dominant genera are characterised as environmental bacteria, with no major pathogens detected amongst the most abundant taxa.

### 3.2 Regional Analysis

**FIGURE 3:**
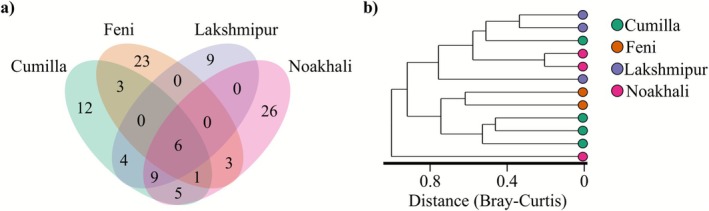
(a) Venn diagram showing the overlap and unique OTUs amongst the four districts. Six OTUs were shared across all regions, whilst Feni and Noakhali exhibited the highest numbers of unique OTUs. (b) Bray–Curtis dendrogram illustrating regional clustering of samples. Most samples grouped by district, though a few outliers were observed in Cumilla and Noakhali. Two broader clusters were evident: Cumilla–Feni and Lakshmipur–Noakhali.

Regional analysis elucidated significant spatial heterogeneity in microbial community composition. A Venn diagram illustrated that only six OTUs were common to all four regions, whilst Feni and Noakhali exhibited the highest number of unique OTUs (23 and 26, respectively), suggesting strong regional specificity (Figure 3a). This finding was corroborated by beta‐diversity analysis, wherein a Bray‐Curtis dendrogram revealed that samples formed distinct clusters primarily based on their geographic origin. Notably, samples from Cumilla and Feni clustered together, whilst those from Lakshmipur and Noakhali formed a separate group, indicating shared microbial profiles between these adjacent districts (Figure 3b).

### 3.3 Pathogen Screening

Metagenomic analysis of floodwater samples revealed distinct geographical patterns of pathogenic bacterial contamination across the study regions (Table 1). In Cumilla, we identified five clinically relevant species, including *Aeromonas dhakensis* and *Mycobacterium bacteremicum*, whilst Feni exhibited the highest microbial diversity with 14 pathogenic species, notably featuring multidrug‐resistant organisms such as *Klebsiella pneumoniae* and *Pseudomonas aeruginosa* . Lakshmipur's profile was dominated by *Acinetobacter* spp. (particularly *A. baumannii* ) and *Clostridium perfringens* . Noakhali demonstrated concerning co‐occurrences of enteric pathogens ( *Escherichia coli* ), emerging *Aliarcobacter* species, and streptococcal variants ( *Streptococcus suis* ).

**TABLE 1:** Pathogenic species identified in floodwater samples across study locations.

Location	Pathogenic species found
Cumilla	*Acinetobacter soli* , *Aeromonas dhakensis*, *A. jandaei* , *Mycobacterium bacteremicum*, *Paracoccus sanguinis*
Feni	*A. soli* , *A. caviae* , *Burkholderia vietnamiensis* , *Enterobacter cloacae* , *E. hormaechei* , *E. roggenkampii*, *Klebsiella pneumoniae* , *K. quasipneumoniae*, *K. variicola* , *Kosakonia radicincitans*, *Phytobacter diazotrophicus*, *P. ursingii*, *Pseudomonas aeruginosa* , *Stenotrophomonas maltophilia*
Lakshmipur	*A. baumannii* , *A. junii* , *A. pittii* , *Clostridium perfringens*
Noakhali	*A. baumannii* , *A. junii* , *A. caviae* , *Aliarcobacter butzleri*, *A. cryaerophilus* , *Escherichia coli* , *K. pneumoniae* , *Prevotella copri* , *Streptococcus pasteurianus* , *S. suis*

### 3.4 AMR Genes, Plasmids and Virulence Factors

Metagenomic analysis revealed antimicrobial resistance (AMR) determinants and virulence potential amongst floodwater pathogens (Table 2). Cumilla exhibited a high prevalence of β‐lactamase genes (*bla*‐A, *bla*VEB) and alginate biosynthesis genes (*algA‐D*), suggesting adaptive advantages in biofilm formation and carbapenem resistance. Feni displayed the most diverse AMR profile, encompassing aminoglycoside (*aac*, *aadA*), sulfonamide (*sul*), and quinolone (*qnrS*) resistance genes, alongside plasmid‐mediated mechanisms (IncP1, Col440I). Notably, hypervirulence factors in Feni included type VI secretion system components (*hsiB1*, *pscF‐G*) and phenazine toxins (*phzA1*), indicative of enhanced pathogenicity. Lakshmipur's AMR burden was very low and dominated by *sul*, whilst Noakhali harboured clinically critical genes (*bla*OXA, *ermB*, *tetM*) and mobilizable plasmids (IncQ1, ColKP3), highlighting the potential for horizontal gene transfer. The conserved presence of *acpXL* (lipid A biosynthesis) and *phoP/relA* (stress response regulators) across all regions suggests core virulence adaptations to floodwater environments.

**TABLE 2:** Distribution of AMR genes, plasmids, and hypervirulence (HV) factors across study locations.

Category	Cumilla	Feni	Lakshmipur	Noakhali
AMR Genes	*bla‐*A, *bla*VEB, *flo, sul*	*aac, aad, ant, bla‐*A, *bla*CMY, *bla*OXA, *bla*PAU, *bla*SHV, *bla*VEB, *cat, cml, dfr, fos, qnr, sul, tet*	*sul*	*aac, aad, ant, aph, bla‐*A, *bla*GES, *bla*OXA, *bla*VEB, *cat, cfx, cml, dfr, ere, erm, lnu, lsa, mef, mph, msr, qnr, sul, tet*
Plasmids	IncP1	Col440I, ColRNAI, IncP1		Col440I, ColKP3, IncP6, IncQ1
HV Factors	*acpXL, alg8, algA, algC, algD, algI, algU, hcp1, icl, phoP, relA, waaF*	*acpXL, chpC, fepC, fimT, flgN, fliL, hsiB1, icl, mbtH, mucE, ompA, pchB, pcrG, phzA1, pscF, pscG, pscL, xcpU, xcpV, yagX, yagY, ykgK*	*acpXL, icl, ideR, phoP, relA*	*acpXL, icl, phoP, relA*

## 4 Discussion

Our metagenomic analysis confirms that floodwaters in Bangladesh carry clinically significant pathogens, with the distribution of this microbial diversity being strongly influenced by regional factors. This concept of landscape epidemiology, where geography dictates disease risk, has long been recognised (Pavlovsky 1966). A key finding was the spatially structured nature of the pathogen load (Xiang et al. 2024; Fan et al. 2024; Oh et al. 2024); for instance, Feni exhibited the highest diversity, including multidrug‐resistant *Klebsiella pneumoniae* and toxigenic *Pseudomonas aeruginosa* , whilst Noakhali revealed the co‐occurrence of enteric pathogens with emerging zoonotic agents. This regional heterogeneity aligns with contemporary studies showing that local anthropogenic activities distinctly shape the water microbiome (Staley et al. 2014). To implement targeted surveillance, we recommend that public health authorities use such spatial data to create risk maps. The specific pathway would involve prioritising districts like Feni and Noakhali for pre‐emptive interventions, such as deploying mobile water testing units and issuing targeted health advisories about specific pathogens (e.g., gastrointestinal risks in Noakhali vs. wound infection risks in Feni) during future floods.

The floodwater resistome revealed a significant capacity for antimicrobial resistance dissemination, primarily driven by mobile genetic elements (Li et al. 2025; Sharif et al. 2023). The critical role of plasmids in the environmental spread of AMR was established decades ago (Levy and Marshall 2004). Our detection of broad‐host‐range plasmids such as IncP1 in Feni, carrying a diverse suite of AMR genes, provides a contemporary and concerning validation of this paradigm in a high‐exposure scenario. Furthermore, the identification of livestock‐associated resistance genes (*tetM*, *ermB*) in Noakhali provides a direct genomic link to agricultural runoff, a major contributor to environmental AMR burdens (Robinson et al. 2016). The lower AMR gene diversity in Lakshmipur may reflect sampling from sites with reduced anthropogenic pressure, lower microbial biomass affecting detection sensitivity, or genuine geographic variation in the resistome. This pattern underscores the spatial heterogeneity of AMR in aquatic and floodwater environments, which has been observed in other regional and global studies (Kim et al. 2022; Delgado‐Baquerizo et al. 2022; Serrana et al. 2025). This finding directly informs a key target of Bangladesh's National Action Plan on AMR: reducing environmental contamination. The planned pathway for implementation involves integrating environmental AMR monitoring into the national framework.

When contextualised within the global literature, our findings both corroborate and extend existing models of flood‐related microbial diversity. The concept of floods as drivers of waterborne disease is well documented (World Health Organization 2018). The abundance of *Aeromonas dhakensis* observed in our samples, for example, supports the modern hypothesis that drought‐to‐flood transitions amplify pathogenic populations (Kim et al. 2022). A key advancement of our work is the detailed, genomic‐level resolution of these pathogens, revealing not just their presence but their mobile genetic arsenal. The implementation target stemming from this is the modernization of disaster response. The specific pathway we envision is the adoption of rapid, portable sequencing technologies (e.g., Oxford Nanopore) by emergency response teams. This would allow for real‐time pathogen and AMR gene detection during a flood event, moving beyond slow, culture‐based methods to generate actionable data within days, not weeks.

Despite these insights, the study carries important limitations. With a relatively small sample size (*n* = 12), statistical power and generalizability are constrained. The absence of time‐series sampling means we cannot infer microbial succession before and after flooding events.

Additionally, the focus on bacterial taxa excludes viral pathogens, such as rotavirus and hepatitis A, which are known to spike in post‐flood scenarios and pose significant health burdens.

Limitations in environmental databases—especially for low‐abundance, uncultured taxa—also introduced classification ambiguities (e.g., genus‐level bin GGB62595). Furthermore, whilst certain species have been flagged as pathogenic, the absence of strain‐level resolution in the analysis restricts the confident identification of whether the detected strains are indeed pathogenic variants or harmless environmental counterparts. These limitations, inherent to snapshot metagenomic studies, define a clear path for future research to build upon this initial characterisation.

This study successfully achieved its primary objectives to comprehensively profile pathogenic bacteria and track AMR genes in the 2024 Bangladesh floods. Our metagenomic exploration provides evidence that floodwaters in Bangladesh carry diverse pathogens—including multidrug‐resistant *Klebsiella pneumoniae* and emerging *Aliarcobacter* spp.—and a complex array of AMR genes on mobile plasmids. The key novel knowledge obtained is the demonstration of distinct, region‐specific microbial diversity profiles. Our findings provide a justification for integrating metagenomic tools into national AMR surveillance and flood response frameworks.

## Author Contributions

M.R. and M.J. developed the protocol, and methodology, conceived and coordinated the study, and reviewed the manuscript. T.R., and M.J. interpreted laboratory data, cleaned and finalised the dataset, performed the descriptive analyses, and prepared the first draft of the manuscript. S.B.M. and N.S.N.S. were involved in the laboratory work, and analysis of laboratory data and provided intellectual input to the manuscript. Z.H.M., M.S.I. and M.M.K. critically reviewed the manuscript and provided intellectual input. All authors reviewed subsequent drafts of the manuscript and approved the final version. All authors had full access to all the data in the study and accepted the responsibility for the integrity of the data, accuracy of the data analysis, and for publication.

## Disclosure

The corresponding author affirms that this manuscript is an honest, accurate, and transparent account of the study being reported; that no important aspects of the study have been omitted; and that any discrepancies from the study as planned (and, if relevant, registered) have been explained.

## Ethics Statement

This environmental study involved floodwater sample collection and analysis, with no human or animal subjects. Ethical approval was not required. Sampling followed local regulations, and permissions were obtained from landowners and authorities.

## Conflicts of Interest

The authors declare no conflicts of interest.

## Data Availability

The data that support the findings of this study are available on request from the corresponding author. The data are not publicly available due to privacy or ethical restrictions.
